# Longitudinal Effects of Herpesviruses on Multiple Cognitive Outcomes in Healthy Elderly Adults

**DOI:** 10.3233/JAD-221116

**Published:** 2023-07-18

**Authors:** Bodil Weidung, Maria Josefsson, Peter Lyttkens, Jan Olsson, Fredrik Elgh, Lars Lind, Lena Kilander, Hugo Lövheim

**Affiliations:** a Department of Public Health and Caring Sciences, Section of Clinical Geriatrics, Uppsala University, Uppsala, Sweden; b Department of Statistics, Umeå School of Business, Economics and Statistics, Umeå University, Umeå, Sweden; c Department of Public Health and Caring Sciences, Uppsala University, Uppsala, Sweden; d Department of Clinical Microbiology, Umeå University, Umeå, Sweden; e Department of Medical Sciences, Acute and Internal Medicine, Uppsala University, Uppsala, Sweden; f Department of Community Medicine and Rehabilitation, Division of Geriatic Medicine, Umeå University, Umeå, Sweden; g Wallenberg Centre for Molecular Medicine, Umeå University, Umeå, Sweden

**Keywords:** Aged 80 and over, Alzheimer’s disease, Apolipoproteins E, cognition disorders, cohort studies, cytomegalovirus, dementia, Herpes simplex, herpesvirus 1 human, neurocognitive disorders

## Abstract

**Background:**

Herpesviruses have been proposed to be involved in Alzheimer’s disease development as potentially modifiable pathology triggers.

**Objective:**

To investigate associations of serum antibodies for herpes simplex virus (HSV)-1 and cytomegalovirus (CMV) and anti-herpesvirus treatment with cognitive outcomes in relation to interactions with *APOE* ɛ4.

**Methods:**

The study included 849 participants in the population-based Prospective Investigation of the Vasculature in Uppsala Seniors study. Cognitive performance at the ages of 75 and 80 years was assessed using the Mini-Mental State Examination (MMSE), trail-making test (TMT) A and B, and 7-minute screening test (7MS).

**Results:**

Anti– HSV-1 IgG positivity was associated cross-sectionally with worse performance on the MMSE, TMT-A, TMT-B, 7MS, enhanced free recall, and verbal fluency tests (*p* = 0.016, *p* = 0.016, *p* < 0.001, *p* = 0.001, *p* = 0.033, and *p* < 0.001, respectively), but not orientation or clock drawing. Cognitive scores did not decline over time and longitudinal changes did not differ according to HSV-1 positivity. Anti– CMV IgG positivity was not associated cross-sectionally with cognition, but TMT-B scores declined more in anti– CMV IgG carriers. Anti– HSV-1 IgG interacted with *APOE* ɛ4 in association with worse TMT-A and better enhanced cued recall. Anti– HSV IgM interacted with *APOE* ɛ4 and anti-herpesvirus treatment in association with worse TMT-A and clock drawing, respectively.

**Conclusion:**

These findings indicate that HSV-1 is linked to poorer cognition in cognitively healthy elderly adults, including impairments in executive function, memory, and expressive language. Cognitive performance did not decline over time, nor was longitudinal decline associated with HSV-1.

## INTRODUCTION

Herpesviruses may be involved in Alzheimer’s disease (AD) development as potentially modifiable triggers of amyloid-β deposition and inflammation [1]. Preclinical and epidemiological findings suggest that herpes simplex virus (HSV)-1 increases the risk of AD development [2–15], especially in genetically susceptible individuals [7, 11], and that anti-herpesvirus treatment has a normalizing effect on this risk [16–20]. AD is characterized by cognitive impairment, which develops insidiously over several years before diagnosis. Whether HSV-1 is also associated with cognitive changes very early in disease processes remains unknown.

HSV-1 and cytomegalovirus (CMV) are members of the Herpesviridae family and cause persistent infection. The prevalence of both infections in adult populations is about 80% [21]. HSV-1 is neurotropic and usually lies dormant in the trigeminal ganglia. Periodic reactivation of HSV-1 infection may be asymptomatic or manifest as skin and mucous-membrane lesions, such as cold sores, or rarely as encephalitis [22]. CMV infection is usually asymptomatic, and this virus may reside in cell types such as epithelial cells, endothelial cells, fibroblasts, circulating leukocytes, and specialized parenchymal cells [23]. With increasing age, the expansion of CMV-specific CD8 + T cells may lead to immunological changes involving the reduction of the naïve T-cell compartment available for immune responses, including those to other pathogens such as HSV-1 [24–26]. Accordingly, previous investigations suggest that CMV modulates the association between HSV-1 and incident AD, potentiating the negative impact of HSV-1 on the AD risk [15]. Further studies are needed to confirm this finding. Polymorphisms in the apolipoprotein E (*APOE*) gene are known to modulate both immune function and susceptibility to infections [5]. The *APOE* ɛ4 allele has been found to potentiate the risk of developing AD [7, 8, 27, 28] and cognitive impairment [29] with HSV-1 carriership in some studies and not in another [4].

Few studies have examined associations between HSV-1 or CMV infection and cognition, and results have been inconsistent, highlighting the difficulty of capturing subtle cognitive changes [4, 8, 29–42]. Study cohorts need to be well controlled, particularly with regard to age, a strong driver of cognitive impairment, and other suspected confounding or interacting factors. To our knowledge, the potential effect of anti-herpesvirus treatment on cognition and the potentially modulating effect of CMV and *APOE* ɛ4 on longitudinal associations between HSV-1 infection and cognitive parameters have not been investigated previously. In addition, the use of multiple cognitive assessments may compensate for the limitations of individual instruments. The aim of this study was to investigate cross-sectional and longitudinal associations of HSV-1 carriership and reactivation, CMV carriership, and anti-herpesvirus treatment, separately, with performance on multiple cognitive assessments in relation to potential interactions with *APOE* ɛ4 among cognitively healthy 75-year-olds in Sweden. Interaction between HSV-1 and CMV carriership was also investigated in association with the cognitive assessments.

## METHODS

### Setting, design, and participants

This study was conducted with longitudinal data from the Prospective Investigation of the Vasculature in Uppsala Seniors (PIVUS) study, which was conducted with 1,016 residents of Uppsala, Sweden. Uppsala hosts a university, and a large proportion of its inhabitants have received higher education [43]. The primary purpose of the PIVUS study was to evaluate vasodilation indicators as predictors of future cardiovascular events in participants aged 70 years at the time of enrollment (April 2001– June 2004). Baseline assessments included venipuncture and self-reports of educational attainment. Educational attainment was initially grouped into three categories (≤9, 10–12, and > 12 years) and later, the two highest categories were merged due to small numbers (≤9 and≥10 years). Self-reported history of stroke, diabetes, and congestive heart failure were collected at each visit. Participants were followed with cognitive assessments performed at the ages of 75 and 80 years and all who underwent cognitive testing were included in the present study. The study was approved by the Regional Ethical Review Board of Uppsala (#2017/349) and the Swedish Ethical Review Authority (#2020-00241, #2020-06029). All participants provided written informed consent.

### Serology and APOE ɛ4 genotyping

The first available serum samples were used in this study. Samples stored at – 80°C were thawed and analyzed for anti–HSV immunoglobulin (Ig) G, anti–HSV IgM, and –CMV IgG seropositivity (carriership) using an in-house enzyme-linked immunosorbent assay (ELISA), modified from that described by Juto and Settergren [44], at the Department of Clinical Microbiology, Section of Virology, Umeå University, Sweden. The Umeå clinical isolate HSV-1 1351-95 and the CMV 169 laboratory strain were used for antigen production, as described previously [2, 45]. Anti– HSV and anti– CMV IgG positivity were defined as≥5% of the net absorbance (absorbance_virus-coated well_ – absorbance_control_) at 405 nm, and anti– HSV IgM positivity was defined as≥0.15% of the net absorbance. The pool of anti– HSV IgG– positive samples were further analyzed down to species-level for presence of anti– HSV-1 IgG using a commercial ELISA kit (HerpeSelect 1; FOCUS Diagnostics, Cypress, CA, USA). All analyses were performed in duplicate.

The *APOE* ɛ4 genotype of PIVUS study participants were determined by the genotyping of single nucleotide polymorphisms rs429358 and rs7412 (ɛ2, ɛ3, ɛ4) at the single nucleotide polymorphism technology platform of Uppsala University, Sweden, using the Golden Gate assay (Illumina Inc., San Diego, CA, USA) [46]. For participants with missing genotype data, serum samples were analyzed to detect the apolipoprotein E4 (apoE4) phenotype using the Human ApoE4 ELISA Kit (BioVision, San Francisco, CA, USA) at the Department of Clinical Microbiology, Umeå University.

### Anti-herpesvirus prescriptions

Data on anti-herpesvirus prescriptions, defined using the anatomical therapeutic chemical classes J05AB01– J05AB20, J05AP01, and J05AD01, were collected from medical records for anti– HSV IgG carriers and complemented with nationwide data on 2005–2019 pharmacy-dispensed prescriptions for the entire cohort from the Swedish Prescribed Drug Register, administered by the National Board of Health and Welfare. This register also includes information on dispensed drugs delivered by mail or proxy or re-packaged as part of a pharmacy dispensing service (ApoDos) commonly used by elderly adults. All prescriptions made before the age of 80 years were included.

The medical record review was performed between May 2020 and February 2021 and included multiple sources. Drug prescriptions made in primary care centers and hospitals in the Uppsala county council from 2005 were extracted from participants’ medical records. Additionally, data on drug prescriptions made before 2005 were collected from a paper archive for participants who were deceased before 2017 (complete prescription histories from primary care and hospital settings) and a digitalized archive including medical records from the earliest record, which was around 1990–1994 (the time of conversion differed among caregivers) for persons alive at the time of data collection or deceased in 2017 or after. For these individuals, prescriptions from before 2005 and from hospitals were accessible only as scanned record notations. A physician reviewed these notations for the first 81 participants in order of inclusion; as no additional prescription was found, this review was discontinued. Medical records predating 2005 from some private caregivers were inaccessible, although this issue was encountered infrequently because private practices were rare in the area at that time. A physician performed all medical record reviews except that of records in the paper archive, which was performed by an archivist.

Suspected treatment indications for anti-herpesvirus drug prescriptions were interpreted in relation to adherence to the manufacturer’s local guidelines for the treatment of HSV and varicella zoster virus infections (being the most common treatment indications), as presented in a national medical drug product register [47]. The number of prescribed doses of each drug was compared to recommended treatment schemes for each treatment indication and in cases of perfect agreement, the treatment indication was interpreted.

### Outcome measures

At the age of 75 years, a trained research nurse administered the Mini-Mental State Examination (MMSE), Trail Making Test (TMT) A and B, and the four brief tests of the 7-minute screening test (7MS) – enhanced free and cued recall, Benton temporal orientation, verbal fluency, and clock drawing. Only the MMSE and the TMT-A and -B were assessed at the age of 80 years. The MMSE is used to assess individuals’ visuospatial, language, concentration, working memory, memory recall, and orientation abilities [48, 49]. Total scores range from 0 to 30, with higher scores indicating greater cognitive ability. The revised Swedish version of the MMSE was administered according to a previously established procedure [50, 51]. The TMT is used to assess executive function, related mainly to the speed and fluidity of cognitive abilities [52, 53]. It showed the greatest prodromal decline among 15 cognitive parameters in persons later diagnosed with AD in a previous study [54], indicating its relevance for the measurement of abilities affected in prodromal AD. For the TMT-A, participants are timed while drawing lines between 25 encircled numbers in numeric order on paper; for the TMT-B, numbers and letters are alternated, offering a greater challenge, primarily in terms of speed. The numbers of seconds required to complete the tests were recorded (TMT-B maximum = 600 s) and treated as continuous variables. The 7MS was developed to distinguish patients with probable AD from cognitively healthy individuals [55], and has been validated also for all-cause dementia [56]. It consists of four brief tests— enhanced free and cued recall, Benton temporal orientation, verbal fluency, and clock drawing— which assess memory, orientation, expressive language, and visuospatial ability, respectively. In the enhanced recall test, individuals are instructed to remember 16 items presented 4 at a time after a distraction activity, with (cued recall) or without (free recall) semantic cues. The number of freely or cued recalled items is counted, with total scores ranging from 0 to 16. In the verbal fluency test, individuals are instructed to generate unique examples of as many animals as possible in 1 min. The score is the total number of examples provided. In the Benton temporal orientation test, participants are asked about the current month, date, year, day of the week, and time of day. Responses are scored relative to the degree of error: 5 points for each month off (maximum = 30), 10 points for each year off (maximum = 60), 1 point for each date off (maximum = 15), 1 point for each weekday off (maximum = 3), and 1 point for each 30-min deviation (maximum = 5). The maximum score is 113, indicating low performance. In the clock drawing test, participants draw the face of a clock with all numbers and the hands indicating “twenty to four.” One point each is given for drawing the numbers 1–12, placing them in the correct order and locations, drawing two hands, indicating the correct hour and minute, and making the hour hand shorter than the minute hand. The maximum score is 7. Total 7MS scores (representing the log-odds of having AD) were calculated using Solomon et al.’s [55] formula: total score = 35.59 – 1.303×ECR – 1.378×VF+3.298×BTO – 0.838×CD, where ECR, VF, BTO, and CD are the scores for enhanced cued recall, verbal fluency, Benton temporal orientation, and clock drawing, respectively.

### Statistical analyses

The χ^2^ test was used to analyze differences in the proportions of women, *APOE* ɛ4 carriers, anti– HSV-1 IgG and anti– CMV IgG carriers, and individuals with low educational attainment between included and not included persons, and patterns of missing data (when > 5%). Correlations between cognitive assessment scores were examined using Spearman’s ρ. Follow-up time was a dichotomous variable differentiating measurements made at the ages of 75 and 80 years.

Linear mixed models were used to investigate the effects of anti– HSV-1 and CMV IgG positivity on MMSE, TMT-A, and TMT-B performance from the assessments conducted at the ages of 75 and 80 years in the full sample, and of anti– HSV IgM positivity and anti-herpesvirus drug use among HSV-1 IgG carriers. The full models included a random intercept and fixed main effects for follow-up time, sex, education, and *APOE* ɛ4, as well as anti– HSV-1 and anti– CMV IgG positivity in the full sample and anti– HSV IgM positivity and anti-herpesvirus drug use in the subsample. Effects of longitudinal changes in main effects on outcomes were examined by including two-way interactions between each main effect and follow-up time.

The final models were extended to search for additional cross-sectional and longitudinal interactions between anti– HSV-1 IgG positivity and *APOE* ɛ4, anti– CMV IgG positivity and *APOE* ɛ4, and anti– HSV-1 IgG positivity and anti– CMV IgG positivity in the full sample, and between anti– HSV IgM positivity and *APOE* ɛ4, anti-herpesvirus drug use and *APOE* ɛ4, and anti– HSV IgM positivity and anti-herpesvirus drug use in the subsample, in separate analyses. Restricted maximum likelihood and type III sums of squares were used in all models.

Using linear regression, similar models were built for the 7MS total and subscale scores from the assessments conducted at the age of 75 years, including sex, education, and *APOE* ɛ4, as well as anti– HSV-1 and anti– CMV IgG positivity in the full sample and anti– HSV IgM positivity and anti-herpesvirus drug use in the subsample.

The normality of all model residuals was examined using the Kolmogorov– Smirnov test; in case of non-normality, transformations were evaluated. Residual weights were included to compensate for any bias due to dropout before cognitive testing at the age of 75 years. The weights were calculated using multiplicative inverse probabilities estimated with logistic regression of missing data for the outcome variable with all predictors and history of diabetes, congestive heart failure, or stroke before the age of 80 years. As a sensitivity analysis, the models were repeated with the inclusion of a composite variable of history of stroke, congestive heart failure, or diabetes before the age of 80 years.

No correction for multiple testing was performed because the statistical analysis followed pre-defined hypotheses [57]. All planned analyses are presented. All tests were two tailed and *p*≤0.05 was considered to reflect significance. The analyses were performed using IBM SPSS Statistics (ver. 28.0.0.0 for Windows; IBM Corporation, Armonk, NY, USA). The matplotlib library (version 3.4.3) in Python (version 3.9.7) was used in the Spyder integrated development environment (version 5.1.5) in Anaconda Navigator (version 2.1.1 for Windows; 2016 Anaconda Inc., Austin, TX, USA) for data visualization.

## RESULTS

**Fig. 1:**
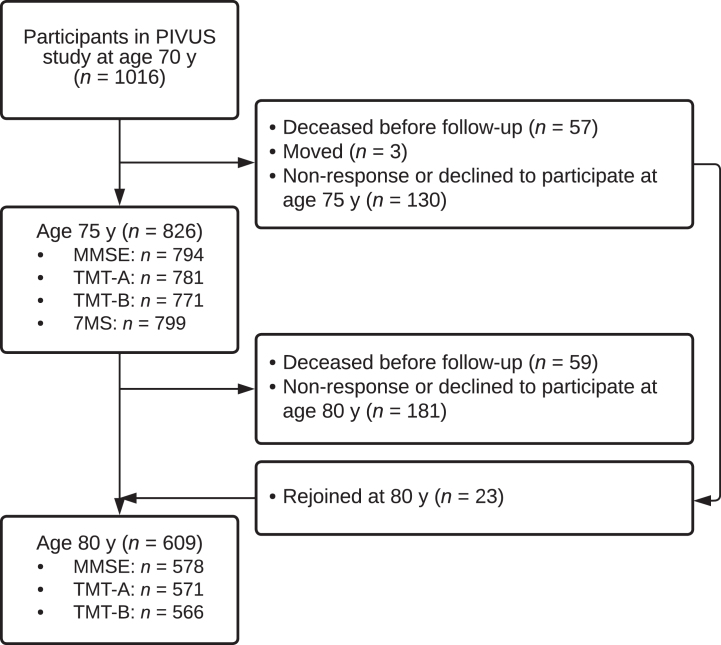
Flow of study participation. PIVUS, Prospective Investigation of the Vasculature in Uppsala Seniors; y, years; MMSE, Mini-Mental State Examination; TMT, trail-making test; 7MS, 7-minute screening test.

The flow of participants is presented in Fig. 1. In total, 849 PIVUS study participants were included in this study; 586 participants underwent cognitive assessment at the ages of 75 and 80 years, 240 underwent assessment only at the age of 75 years, and 23 underwent assessment only at the age of 80 years. For 6 participants, sera sampled at the age of 75 years were used since samples from the age of 70 years were missing. Serum samples from 80 participants were analyzed for apoE4 phenotype due to missing *APOE* ɛ4 genotype. Congruence between *APOE* ɛ4 genotyping and apoE4 phenotyping was absolute for 23 participants whose samples were both geno- and phenotyped. The characteristics of the sample are presented in Table 1. The prevalence of anti– HSV-1 IgG carriers was 75%. The overall anti-herpesvirus drug prescription frequency was 5.8% ; 6.0% among anti– HSV-1 IgG carriers and 5.2% among non-carriers (*n* = 49, 38, and 11, respectively). Among carriers of anti– HSV IgG, which is non-specific to HSV subtype, the prescription frequency was 6.4% and among non-carriers 2.7% (*n* = 45 and 4, respectively). First prescriptions were found between June 28, 1991 and April 1, 2008 (median = August 13, 2003; Q1 = October 1, 1999; Q3 = July 15, 2006). The baseline cognitive assessments were made between April 3, 2006 and August 18, 2009 (median = January 23, 2008; Q1 = April 27, 2007; Q3 = September 9, 2008). All first anti-herpesvirus prescriptions were made before the first cognitive assessment (median time to assessment = 5.36 years, inter-quartile range = 15.9 years). Differences between included and not included participants and patterns of missing data are presented in the Supplementary Material (Supplementary Results).

**Table 1:** Characteristics of the sample

Characteristic	*N*	Prevalence
Sex, female, *n* (%)	849	432 (50.9)
Highest education, *n* (%)	841
≤9 y	458 (54.5)
≥10 y	383 (45.5)
*APOE* ɛ4+, *n* (%)	848	241 (28.4)
Anti-HSV-1 IgG+, *n* (%)	848	636 (74.9)
Systemic anti-herpesvirus treatment, *n* (%)	636	38 (6.0)
Anti-HSV IgM+, *n* (%)	636	71 (11.2)
Anti-CMV IgG+, *n* (%)	848	669 (78.9)
Diabetes, *n* (%)	848	122 (14.4)
Congestive heart failure, *n* (%)	848	93 (11.0)
Stroke, *n* (%)	848	103 (12.1)

HSV, herpes simplex virus; Ig, immunoglobulin; CMV, cytomegalovirus.

**Fig. 2:**
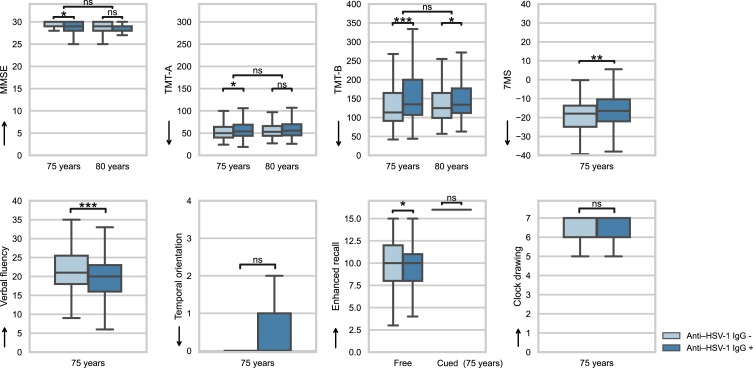
Distribution of cognitive outcomes with respect to anti– HSV-1 IgG positivity. HSV-1, herpes simplex virus type 1; IgG, immunoglobulin G; MMSE, Mini-Mental State Examination; TMT, trail-making test; 7MS, 7-minute screening test. Arrows indicate the direction of greater cognitive ability. Significance testing was performed using multiple mixed and linear regression. *p≤0.05, **p≤0.01, ***p≤0.001; ns, not significant.

Figure 2 shows distributions of cognitive outcomes with respect to anti– HSV-1 IgG positivity. Generally, participants performed well on the cognitive tests and HSV-1 carriers performed slightly worse than did non-carriers. Variations in MMSE, enhanced cued recall, clock drawing, and Benton temporal orientation scores were small, with marked ceiling effects observed for the three former scores and a floor effect observed for the latter. All cognitive scores from assessments performed at the age of 75 years correlated (*p*≤0.001), except that the Benton temporal orientation and clock drawing scores did not correlate with each other (Supplementary Table 1). In the sample of participants who underwent both assessments, absolute longitudinal differences between MMSE scores and TMT-A and TMT-B completion times obtained at the ages of 75 and 80 years correlated (TMT-A with TMT-B or MMSE: *p*≤0.001, TMT-B with MMSE: *p*≤0.05; Supplementary Table 2).

**Table 2:** Mixed and linear regression results for cognitive outcomes with anti– HSV-1 IgG+and anti– CMV IgG+

	MMSE (*n*: T1 = 794, T2 = 578)		TMT-A (*n*: T1 = 781, T2 = 571)		TMT-B (*n*: T1 = 771, T2 = 566)		7MS total score (*n*: T1 = 799)	
	β (95% CI)	*p*	β (95% CI)	*p*	β (95% CI)	*p*	β (95% CI)	*p*
Cross-sectional
Anti– HSV-1 IgG+	-0.29 (-0.53– -0.05)	0.016	4.92 (0.90–8.94)	0.016	30.84 (13.23–48.44)	<0.001	4.78 (1.93–7.63)	0.001
Anti– CMV IgG+	-0.06 (-0.27–0.26)	0.963	0.31 (-4.25–4.87)	0.893	-3.10 (-22.39–16.19)	0.753	-0.99 (-4.32–2.34)	0.558
Anti– HSV-1 IgG+×*APOE* ɛ4	-0.08 (-0.54–0.38)	0.732	7.85 (0.33–15.38)	0.041	15.61 (-20.84–52.07)	0.401	4.41 (-2.22–11.04)	0.192
Anti– CMV IgG+×*APOE* ɛ4	-0.11 (-0.62–0.40)	0.673	-3.54 (-11.91–4.82)	0.406	-7.49 (-46.75–31.76)	0.708	-1.34 (-8.89–6.22)	0.728
Anti– HSV-1 IgG+×anti– CMV IgG+	-0.31 (-0.79–0.17)	0.200	0.34 (-7.61–8.30)	0.933	29.12 (-9.08–67.33)	0.135	-0.78 (-7.59–6.03)	0.822
Longitudinal
Anti– HSV-1 IgG+	0.15 (-0.14–0.43)	0.317	-0.87 (-6.01–4.26)	0.739	-9.92 (-23.45–3.61)	0.150	N/A
Anti– CMV IgG+	-0.17 (-0.50–0.17)	0.331	2.17 (-3.90–8.25)	0.482	17.92 (1.96–33.88)	0.028	N/A
Anti– HSV-1 IgG+×*APOE* ɛ4	-0.35 (-1.01–0.31)	0.301	3.99 (-7.71–15.68)	0.504	25.48 (-5.53–56.49)	0.107	N/A
Anti– CMV IgG+×*APOE* ɛ4	0.11 (-0.65–0.87)	0.771	-1.79 (-15.27–11.69)	0.794	18.16 (-17.82–54.15)	0.322	N/A
Anti– HSV-1 IgG+×anti– CMV IgG+	-0.31 (-0.99–0.38)	0.380	-7.08 (-19.41–5.25)	0.260	-9.23 (-41.51–23.04)	0.574	N/A

HSV-1, herpes simplex virus type 1; CMV, cytomegalovirus; IgG, immunoglobulin G; MMSE, Mini-Mental State Examination; TMT, trail-making test; 7MS, 7-minute screening test; CI, confidence interval. T1 and T2 denotes the data collections performed at 75 and 80 years. Mixed models were used for MMSE, TMT-A, and TMT-B scores, and linear regression was performed for 7MS scores. Mixed models included a fixed and random intercept and were adjusted for anti– HSV-1 IgG, anti– CMV IgG, *APOE* ɛ4, sex, education, follow-up time, and two-way interactions between each main effect and follow-up time. The linear regression model included intercept, anti– HSV-1 IgG, anti– CMV IgG, *APOE* ɛ4, sex, and education. Additional interactions were added in separate models.

### Associations between anti– HSV-1 IgG positivity and cognition in the full sample

Tables 2 and 3 show the results from the final models of associations of cognitive outcomes with anti– HSV-1 and anti– CMV IgG positivity, respectively. Anti– HSV-1 IgG positivity was associated cross-sectionally with worse performance on the MMSE, TMT-A, TMT-B, total 7MS, enhanced free recall, and verbal fluency tests (*p* = 0.016, *p* = 0.016, *p* < 0.001, *p* = 0.001, *p* = 0.033, and *p* < 0.001, respectively). Anti– CMV IgG carriership did not affect the cross-sectional cognitive outcomes.

**Table 3:** Linear regression results for 7MS subscales with anti– HSV-1 IgG+and anti– CMV IgG+at the age of 75 years

	Clock drawing (*n* = 785)		Enhanced free recall (*n* = 790)		Enhanced cued recall (*n* = 790)		Verbal fluency (*n* = 787)		Temporal orientation (*n* = 792)	
	β (95% CI)	*p*	β (95% CI)	*p*	β (95% CI)	*p*	β (95% CI)	*p*	β (95% CI)	*p*
Main effects
Anti– HSV-1 IgG+	-0.11 (-0.25–0.04)	0.145	-0.40 (-0.77– -0.03)	0.033	-0.03 (-0.17–0.11)	0.652	-1.80 (-2.71– -0.88)	<0.001	0.68 (-0.04–1.39)	0.063
Anti– CMV IgG+	-0.02 (-0.19–0.15)	0.818	0.20 (-0.23–0.63)	0.369	0.07 (-0.09–0.23)	0.414	-0.21 (-1.28–0.85)	0.694	-0.36 (-1.19–0.47)	0.400
Interactions
Anti– HSV-1 IgG+×*APOE* ɛ4	0.07 (-0.26–0.40)	0.668	-0.30 (-1.16–0.56)	0.489	0.33 (0.01–0.65)	0.046	-0.54 (-2.67–1.58)	0.616	1.28 (-0.37–2.92)	0.128
Anti– CMV IgG+×*APOE* ɛ4	-0.22 (-0.49–0.16)	0.254	0.77 (-0.21–1.74)	0.124	0.09 (-0.28–0.45)	0.650	-0.03 (-2.45–2.38)	0.978	-0.42 (-2.30–1.46)	0.662
Anti– HSV-1 IgG+×anti– CMV IgG+	-0.01 (-0.35–0.33)	0.976	-0.12 (-1.00–0.76)	0.790	0.04 (-0.29–0.38)	0.793	-0.36 (-2.54–1.82)	0.744	-0.37 (-2.07–1.33)	0.668

7MS, 7-minute screening test; HSV, herpes simplex virus; Ig, immunoglobulin; CMV, cytomegalovirus; CI, confidence interval. The models included intercept, anti– HSV-1 IgG, anti– CMV IgG, *APOE* ɛ4, sex, and education. Interactions were added in separate models.

None of the longitudinal cognitive outcomes declined over time and anti– HSV-1 IgG positivity was not associated with change over time in the longitudinal outcomes. Anti– CMV IgG seropositivity was associated only with an increase over time in the TMT-B score, reflecting worse performance (*p* = 0.028).

Interactions between *APOE* ɛ4, anti– HSV-1, anti– CMV IgG positivity, and follow-up time were generally not related to cognitive outcomes or changes therein over time, except that anti– HSV-1 IgG positivity interacted with *APOE* ɛ4 carriership to yield a longer TMT-A completion time and higher scores on the enhanced cued recall test, cross-sectionally (*p* = 0.041 and *p* = 0.046).

The models adjusted for a composite variable of history of stroke, congestive heart failure, or diabetes produced essentially the same results.

### Associations of anti– HSV-1 IgM positivity and anti-herpesvirus drug use with cognitive performance in anti– HSV-1 IgG carriers

Relative to anti-herpesvirus drug prescriptions collected from the register, those collected from medical records indicated earlier dates of first prescriptions, but no additional individual was found to have received such a prescription. The numbers of anti-herpesvirus drug prescriptions adhering to the manufacturer’s recommendations for HSV and varicella zoster virus symptoms among HSV-1 carriers were 7 (18.4%) and 10 (26.3%), respectively; 21 of the 38 prescriptions were uninterpreted.

Neither anti-herpesvirus drug use nor anti– HSV IgM seropositivity was associated with the cognitive outcomes or the change therein over time (Supplementary Tables 3 and 4) in the subsample of anti– HSV-1 IgG carriers. Anti– HSV IgM positivity interacted with anti-herpesvirus drug use to yield a lower score on clock drawing (*p* = 0.034) and with *APOE* ɛ4 carriage to yield a longer TMT-A completion time, i.e., worse cognitive performance, at 75 years (*p* = 0.031). No other interaction between follow-up time, *APOE* ɛ4 carriage, anti– HSV IgM positivity, or anti-herpesvirus drug use was found. Details of model performance are presented in the Supplementary Material.

## DISCUSSION

In this cohort of elderly adults with strong cognitive performance, anti– HSV-1 IgG carriage was associated cross-sectionally with worse performance on MMSE, TMT-A, TMT-B, 7MS, enhanced free recall, and verbal fluency, indicating that HSV-1 affected executive function, memory, and expressive language. Cognitive scores did not change between the ages of 75 and 80 years, reducing the ability to detect longitudinal associations, and longitudinal changes did not differ between HSV-1 carriers and non-carriers.

Previous studies of cross-sectional associations between HSV IgG positivity and cognition have produced mixed results [4, 8, 29–37, 39], possibly due in part to the etiological heterogeneity of cognitive impairment, which can result from a number of diseases or simply from aging. Cognitive test scores are also affected by several cohort-related factors, such as age and education, which may be difficult to control with adjustment. The present study was conducted with a relatively homogenous sample of cognitively healthy individuals of the same age, eliminating some of these factors, but also reducing the comparability of the results with those of other studies.

The TMT-A, TMT-B, enhanced free recall, and verbal fluency assessments, which were affected by HSV-1 carriership in this study, may assess executive function, memory, and expressive language [52, 53, 55]. These functions may be impaired in individuals with early AD [54, 58]. MMSE was also affected by HSV-1 and may assess vocabulary, reasoning, and memory, although their respective contributions may be age dependent and may shift from primarily reasoning to memory around the age of 70 years [49]. This shift may be impacting the validity and comparability of MMSE results between cohorts of different ages, possibly explaining some of the diversity among previous results. Total 7MS scores (which were also affected by HSV-1 carriership) were largely influenced by verbal fluency scores (likely because of the large degree of variation therein), which correlated strongly.

No longitudinal cognitive change between the ages of 75 and 80 years was seen in association with HSV-1, in line with most previous findings [4, 31, 33, 35, 37, 39]. Longitudinal associations were found in the Betula study [8] between anti– HSV IgG positivity and episodic memory decline and in the Rotterdam study [38] between anti– HSV-1 IgG positivity and declines in global cognition, memory, information processing, and executive function, but not with declines in motor function or the MMSE score. Larger average cognitive declines than observed in our sample may be needed to detect such effects. Selective attrition may also have limited the ability to detect such associations, as HSV-1 carriers were less likely to participate in the follow-up assessment.

CMV carriership was not associated with cognitive performance at the age 75, in line with most previous findings [34, 35, 40–42], with one exception [31]. However, TMT-B performance declined more rapidly among CMV carriers than among non-carriers in the present study. TMT-B is a challenging test of executive function, and the observed effect may be specific to this domain or detectable only with more challenging tests. Declines in TMT performance have been linked to prodromal AD [54]. Previous studies of longitudinal associations of CMV carriership with cognition have yielded inconsistent results [31, 35, 37, 39, 40]. No previous study has involved adjustment for HSV-1 carriership, and HSV-1 has been found to interact with CMV in association with incident AD and thus possibly also in association with cognitive impairment [15]. In the present study, the interaction between CMV and HSV-1 IgG seropositivity was not replicated with the use of measures of early cognitive impairment, suggesting that it has no effect or only a subtle effect in individuals with strong cognitive performance. However, the lack of adjustment for HSV-1 carriership in previous studies may have resulted in CMV carriership’s reflection of unaccounted-for main effects of HSV-1 on cognition (i.e., confounding).

The prevalence of anti-herpesvirus drug use was in line with a previous study of a Swedish cohort [16] and slightly lower than in a French database [20]. Among HSV-1 carriers in our sample, anti-herpesvirus drug use was not associated with very early cognitive changes, except in interaction with HSV IgM carriage, which was associated with worse performance on the clock drawing test. This finding may indicate that potential benefits of anti-herpesvirus treatment (previously observed for the AD risk [16–20]) are mainly not captured by early changes in cognitive performance, or that more sensitive cognitive evaluations are needed. As information on treatment indications was unavailable for our sample, we do not know whether treatments were received for HSV-1 or other herpesvirus symptoms, such as shingles; this lack of knowledge compromises the validity of the treatment data. Anti-herpesvirus drug prescriptions were more common among HSV carriers than among non-carriers, suggesting that a portion of HSV carriers received treatment for HSV symptoms. The interpretation of suspected treatment indications based on the adherence of prescription dosages to the manufacturer’s guidelines revealed that a portion of prescriptions likely were issued for HSV symptoms and not all treatment effects should be attributed to VZV. Future studies could examine anti-herpesvirus treatment in relation to verified HSV reactivation to better distinguish potential treatment effects.

Anti– HSV IgM positivity was not associated with cognitive performance among HSV-1 carriers in our sample, with the exception that its interaction with *APOE* ɛ4 was associated with lower performance on the TMT-A assessment and the previously mentioned interaction with anti-herpesvirus drug use in association with poorer performance on the clock drawing test. Two previous studies revealed longitudinal associations between HSV IgM positivity and cognitive decline (episodic memory and MMSE scores) [4, 8]. However, as the present analyses were performed among HSV-1 IgG carriers to distinguish the effect of IgM from that of IgG seropositivity, the results may not be comparable to previous findings. Furthermore, IgM may have poor specificity for the detection of reactivation, as previously argued [59], placing a greater demand on the statistical power to detect differences between groups.

An interaction effect with *APOE* ɛ4 was observed for anti– HSV-1 IgG and anti– HSV IgM carriage, separately, in association with worse performance on the TMT-A in our sample, suggesting that these factors have a synergistic effect on executive dysfunction, as previously seen for declines in episodic memory and MMSE scores, and for the risk of AD [7, 8, 27–29]. An interaction between *APOE* ɛ4 and anti– HSV-1 IgG was also observed in association with better performance on the enhanced cued recall. These interactions were not consistent across cognitive outcome measures, possibly reflecting less robust effects. However, the small variations in many variables and the ceiling and floor effects may have impaired the ability of several assessments to detect minor cognitive variations.

Overall, this study found small but significant effects of HSV-1 across a range of cognitive measures among elderly adults with strong cognitive performance. Together with the accumulating evidence for an association between HSV-1 and AD, this indicates a role of HSV-1 early in the neurodegenerative process and highlights its importance as a risk factor. It may also suggest that the effects of future prevention trials potentially could be detected early in the disease progression, using cognitive measures. Additional studies are needed to determine the value of targeting suspected risk groups, such as risk gene carriers, and the optimal timing of interventions for such trials.

The strengths of the present study include the examination of a large population-based sample and the contemporary nature of the cohort, ensuring its populational relevance. The study was well controlled and the use of data from individuals of the same age eliminated the impact of age on the studied associations. The longitudinal design enabled the investigation of cognitive changes, and the use of multiple cognitive assessments provided a broad perspective on participants’ cognition. Study limitations include the underrepresentation of persons with diabetes, congestive heart failure, and stroke in the PIVUS cohort [60]. Additionally, persons with lower educational attainment and *APOE* ɛ4 carriership were underrepresented among PIVUS study participants who underwent cognitive assessment, likely due to survival bias (as participants were included 5 years after baseline at the earliest), rendering the results less generalizable to those groups. The same factors may have led to the underrepresentation of cognitively impaired individuals, as suggested by the participants’ relatively high cognitive performance. However, 72.8%, 73.1%, and 73.4% of participants underwent follow-up MMSE, TMT-A, and TMT-B assessment, respectively, which may be rather normal for this age group. Missing MMSE and TMT scores were overrepresented among persons with lower educational attainment and anti– HSV-1 IgG positivity, rendering the results less generalizable to those populations. Selective attrition is likely related to survival and health status, and the longitudinal analyses performed in this study may thus have been affected by survival bias, with the potential underestimation of any trends toward cognitive decline. However, the cognition level, rather than the rate of change, has been shown to influence attrition in longitudinal studies [61]. Most cognitive measures correlated in this study, and more extensive cognitive evaluation is needed to determine the impacts of HSV-1 carriership on specific cognitive domains. As associations with more advanced cognitive impairment were not evaluated, the results are generalizable only to populations with strong cognitive performance. Variations in outcomes were small, possibly due to the high level of educational attainment in this cohort and the participation of a large proportion of persons with preserved cognition. The small variations may have impeded the detection of subtle associations in this sample. Ceiling and floor effects were found for several assessments and tests of normality were significant. Finally, information on treatment indications for anti-herpesvirus drug prescriptions was lacking.

### Conclusions

In this population-based cohort of same-aged individuals with strong cognitive performance, HSV-1 carriers performed worse than non-carriers on multiple assessments of cognitive performance, including assessments of executive function, memory, and expressive language at the age of 75 years, indicating robust effects of HSV-1 on cognitive functions that are commonly impaired in early AD. Larger variations in cognitive performance or more precise cognitive measures may be needed to detect the potential influences of CMV carriage, anti– HSV IgM positivity, and anti-herpesvirus treatment on cognitive performance.

## Supplementary Material

Supplementary Material

## Data Availability

The data supporting the findings of this study are available on reasonable request from the corresponding author. The data are not publicly available due to privacy or ethical restrictions.
