# Supplementary material for: Longitudinal Effects of Herpesviruses on Multiple Cognitive Outcomes in Healthy Elderly Adults
Source: J Alzheimers Dis. 2023 Jul 18;94(2):751–62. doi: 10.3233/JAD-221116 (PMC10357165; doi:10.3233/JAD-221116)
Supplement: Supplementary Material [file jad-94-jad221116-s001.pdf]

Supplementary Material

Longitudinal Effects of Herpesviruses on Multiple Cognitive Outcomes in Healthy Elderly Adults

SUPPLEMENTARY RESULTS

Differences between included and not included participants

Among persons not included in this study, who participated only at the age of 70 years when no cognitive testing was performed, lower education attainment (54.5% versus 68.3%, $p < 0.001$) and *APOE* ε4 positivity (28.4% versus 37.0%, $p = 0.002$) were more prevalent, relative to participants. The proportions of women and anti–HSV-1 and anti–CMV IgG carriers did not differ.

Missing values

Missing values (>5%) were found for all outcome variables (MMSE, 19.2%; TMT-A, 20.4%; TMT-B, 21.3%; 7MS, 7.3%). Persons with missing MMSE, TMT-A, or TMT-B values were generally older, and low educational attainment and anti–HSV-1 IgG positivity were more prevalent in this group than among those without missing values: proportions of persons aged 80 years was 83.1% among persons with missing MMSE versus 16.9% among persons with MMSE ($p < 0.001$), 80.3% among persons with missing TMT-A versus 19.7% among persons with TMT-A ($p < 0.001$), and 78.4% among persons with missing TMT-B versus 21.6% among persons with TMT-B ($p < 0.001$); proportions of persons with education ≤ 9 years was 61.3% among persons with missing MMSE versus 52.8% among persons with MMSE ($p = 0.006$), 60.1% among persons with missing TMT-A versus 53.0% among persons with TMT-A ($p = 0.02$), and 60.1% among persons with missing TMT-B versus 52.9% among persons with TMT-B ($p = 0.02$); proportions of persons with anti–HSV-1 IgG positivity was 80.9% among persons with missing MMSE versus 73.6% among persons with MMSE ($p = 0.007$), 81.1% among persons with missing TMT-A versus 73.4% among persons with TMT-A ($p = 0.003$), and 81.9% among persons with missing TMT-B versus 73.1% among persons with TMT-B ($p = 0.001$). No difference was found between these groups in sex, *APOE* ε4 carriership, or anti–CMV positivity. No difference in sex, education, *APOE* ε4, anti–HSV-1 IgG positivity, or anti–CMV IgG positivity was found between participants with and without missing 7MS values.

Missing values did not exceed 5% among predictors (sex, education; *APOE* ε4*;* anti–HSV-1 IgG, anti–CMV IgG, or anti–HSV IgM positivity; or anti-herpesvirus drug use), and patterns thereof were thus not investigated. No imputation was made.

Model performance

For the full sample, the full models (including the full set of covariates and their interactions with follow-up time) performed better than intercepts-only models (those without covariates), indicating that a significant amount of variance in the outcomes was explained by the covariates and interactions (MMSE: $\chi^2 = -101.90$, df = 11, $p < 0.001$; TMT-A: $\chi^2 = -87.20$, df = 11, $p < 0.001$; TMT-B: $\chi^2 = -124.05$, df = 11, $p < 0.001$).

In the HSV-1–positive subsample, the full models performed better than intercepts-only models (MMSE: $\chi^2 = -140.94$, df = 11, $p < 0.001$; TMT-A: $\chi^2 = -181.82$, df = 11, $p < 0.001$; TMT-B: $\chi^2 = -266.56$, df = 11, $p < 0.001$).

Kolmogorov–Smirnov tests of normality were significant for all outcome variables and model residuals ($p \leq 0.001$), indicating remaining within-group variance. The transformation of outcome variables (to logs and square roots except for the 7MS total score, for which log(7MS + $7MS_{min}$ + 1 was used) did not improve the normality of residuals and was thus not used.

Supplementary Table 1. Correlations between cognitive scores at age 75 years

	MMSE	TMT-A	TMT-B	7MS	Temporal orientation	Clock drawing	Verbal fluency	Enhanced free recall
TMT-A	-0.235***							
TMT-B	-0.379***	0.597***						
7MS	-0.416***	0.345***	0.444***					
Temporal orientation	-0.261***	0.139***	0.178***	0.408***				
Clock drawing	0.206***	-0.234***	-0.231***	-0.234***	-0.022			
Verbal fluency	0.341***	-0.308***	-0.412***	-0.928***	-0.129***	0.156***		
Enhanced free recall	0.302***	-0.187***	-0.239***	-0.323***	-0.131***	0.119***	0.295***	
Enhanced cued recall	0.284***	-0.130***	-0.192***	-0.287***	-0.117***	0.158***	0.200***	0.380***

Correlations were tested using Spearman's ρ. MMSE, Mini-Mental State Examination; TMT, trail-making test; 7MS, 7-minute screening test.

*$p \leq 0.05$, ***$p \leq 0.001$.

Supplementary Table 2. Correlations between absolute changes in cognitive scores between ages 75 and 80 years

	MMSE change	TMT-A change
TMT-A change, rho (*n*)	-0.163*** (541)	
TMT-B change, rho (*n*)	-0.137* (534)	0.213*** (533)

Correlations were tested using Spearman's ρ. MMSE, Mini-Mental State Examination; TMT, trail-making test.

*$p \leq 0.05$, ***$p \leq 0.001$.

Supplementary Table 3. Mixed and linear regression results for cognitive outcomes with anti–HSV IgM+ and anti-herpesvirus drug use among anti–HSV-1 IgG carriers

	MMSE (*n*: T1=590, T2=419)		TMT-A (*n*: T1=579, T2=413)		TMT-B (*n*: T1=569, T2=408)		7MS total score (*n*: T1=578)	
	β (95% CI)	*p*	β (95% CI)	*p*	β (95% CI)	*p*	β (95% CI)	*p*
Cross-sectional								
Anti–HSV IgM+	0.15 (-0.22–0.52)	0.415	1.34 (-5.18–7.86)	0.687	-22.68 (-52.91–7.54)	0.141	-2.12 (-6.90–2.66)	0.384
Anti-herpesvirus drugs	-0.16 (-0.64–0.31)	0.500	-0.69 (-10.06–8.68)	0.886	11.15 (-30.30–52.60)	0.597	-2.68 (-9.02–3.65)	0.406
Anti–HSV IgM+ × *APOE* ε4	-0.17 (-1.06–0.72)	0.708	16.59 (1.50–31.69)	0.031	27.57 (-47.24–102.38)	0.469	-0.48 (-13.50–12.54)	0.943
Anti-herpesvirus drugs × *APOE* ε4	-0.97 (-2.15–0.20)	0.104	4.20 (-17.17–25.56)	0.700	-21.05 (-123.16–81.07)	0.686	-4.33 (-24.17–15.51)	0.668
Anti–HSV IgM+ × anti-herpesvirus drugs	0.97 (-0.18–2.13)	0.099	-1.27 (-19.38–21.91)	0.904	16.72 (-91.43–124.86)	0.761	0.07 (-15.97–15.83)	0.994
Longitudinal								
Anti–HSV IgM+	-0.06 (-0.52–0.40)	0.791	-2.39 (-11.18–6.41)	0.594	-4.29 (-29.83–21.25)	0.741	N/A	
Anti-herpesvirus drugs	-0.01 (-0.56–0.59)	0.963	1.48 (-11.12–14.08)	0.818	-8.21 (-43.05–26.63)	0.643	N/A	
Anti–HSV IgM+ × *APOE* ε4	0.21 (-1.34–1.75)	0.793	-18.45 (-46.38–9.48)	0.195	-27.45 (-112.24–57.34)	0.525	N/A	
Anti-herpesvirus drugs × *APOE* ε4	-1.52 (-3.17–0.12)	0.070	-1.28 (-35.65–33.08)	0.942	48.43 (-45.50–142.36)	0.311	N/A	
Anti–HSV IgM+ × anti-herpesvirus drugs	-0.30 (-1.71–1.10)	0.671	11.09 (-19.55–41.74)	0.477	-33.12 (-120.80–54.56)	0.458	N/A	

HSV, herpes simplex virus; Ig, immunoglobulin; MMSE, Mini-Mental State Examination; TMT, trail-making test; 7MS, 7-minute screening test; CI, confidence interval.

T1 and T2 denotes the data collections performed at 75 and 80 years.

Mixed models were used for MMSE, TMT-A, and TMT-B scores and linear regression was performed for 7MS scores. Mixed models included a fixed and random intercept and were adjusted for anti–HSV IgM, anti-herpesvirus drugs, *APOE* ε4, sex, education, follow-up time, and two-way interactions between each main effect and follow-up time. The linear regression model included intercept, anti–HSV IgM, anti-herpesvirus drugs, *APOE* ε4, sex, and education. Additional interactions were added in separate models.

Supplementary Table 4. Linear regression results for 7-minute screening test subscales with anti–HSV IgM+ and anti-herpesvirus drug use among anti–HSV-1 IgG carriers at the age of 75 years

	Clock drawing (n = 582)		Enhanced free recall (n = 586)		Enhanced cued recall (n = 586)		Verbal fluency (n = 585)		Temporal orientation (n = 588)	
	β (95% CI)	p	β (95% CI)	p	β (95% CI)	p	β (95% CI)	p	β (95% CI)	p
Main effects										
Anti–HSV IgM+	0.12 (-0.09–0.33)	0.266	0.26 (-0.28–0.80)	0.342	-0.01 (-0.17–0.19)	0.888	-0.22 (-1.51–1.07)	0.738	-0.22 (-1.51–1.07)	0.738
Anti-herpesvirus drugs	-0.19 (-0.47–0.94)	0.193	-0.01 (-0.72–0.71)	0.993	0.06 (-0.18–0.29)	0.646	0.49 (-1.23–2.20)	0.576	0.49 (-1.23–2.20)	0.576
Interactions										
Anti–HSV IgM+ × *APOE* ε4	-0.31 (-0.88–0.27)	0.293	-0.22 (-1.69–1.25)	0.766	-0.33 (-0.82–0.16)	0.187	-2.12 (-5.64–1.39)	0.236	-1.23 (-4.61–2.15)	0.476
Anti-herpesvirus drugs × *APOE* ε4	0.11 (-0.77–0.99)	0.809	-0.83 (-3.07–1.42)	0.470	-0.19 (-0.94–0.55)	0.610	-1.08 (-6.45–4.29)	0.693	-1.84 (-6.99–3.31)	0.483
Anti–HSV IgM+ × anti-herpesvirus drugs	-0.76 (-1.46–-0.06)	0.034	-0.97 (-0.83–2.76)	0.292	-0.31 (-0.28–0.91)	0.303	1.62 (-2.68–5.92)	0.460	0.55 (-3.58–4.68)	0.795

HSV, herpes simplex virus; Ig, immunoglobulin; CI, confidence interval.
The models included intercept, anti–HSV IgM, anti-herpesvirus drugs, *APOE* ε4, sex, and education. Interactions were added in separate models.
